# Renting vs. Owning: Public Stereotypes of Housing Consumption Decision From the Perspective of Confucian Culture: Evidence From Event-Related Potentials

**DOI:** 10.3389/fpsyg.2022.816004

**Published:** 2022-04-28

**Authors:** Xiaojun Liu, Mingqi Yu, Baoquan Cheng, Hanliang Fu, Xiaotong Guo

**Affiliations:** ^1^School of Management, Xi’an University of Architecture and Technology, Xi’an, China; ^2^Department of Architecture and Civil Engineering, City University of Hong Kong, Kowloon, Hong Kong SAR, China; ^3^Laboratory of Neuromanagement in Engineering, Xi’an University of Architecture and Technology, Xi’an, China

**Keywords:** housing tenure choice, stereotypes, Confucian culture, event-related potentials, implicit attitudes

## Abstract

The ideas of face consciousness, group conformity, extended family concept, and crisis consciousness in Confucian culture have a subtle and far-reaching impact on housing consumption decision among the Chinese public, forming a housing consumption model of “preferring to own a house rather than rent one.” The poor interaction between the housing rental market and the sales market caused by the shortage of rental demand and irrational purchasing behaviors has led to soaring house prices and imbalance between supply and demand that prevail in major cities in China. To gain a deeper understanding of public cognitive attitude toward decisions on owning and renting a house, this study divided the subjects into high and low impact groups based on the overall Confucian culture and four subdimensions. It attempts to take a cognitive neuroscience approach for assessing public stereotypes of housing consumption decision with different types based on the analysis of event-related potentials (ERPs). The results are as follows. First, overall, there is an obvious implicit stereotype of renting a house and explicit stereotype of owning a house among the public. Second, ERPs data show that descriptions of renting a house with positive adjectives could evoke more significant N400 responses. In other words, in the heuristic system, the public perceive that renting a house is restrictive, stressful, unhappy, and crisis. Data from subjective reports show that, after processing information in the analytic system, the public tend to think that owning a house is self-contained, restful, warm, and comfortable. Third, a more negative stereotype of renting a house exists in the high Confucian culture influence group (HIC) Group than in the low Confucian culture influence group (LIC) Group, and is more inclined to own a home. Fourth, under the Confucian culture sub-dimension, there are differences in housing consumption stereotypes between high and low groups in terms of extended family concept, group conformity, and crisis consciousness. Fifth, the moderating effect analysis found that perceived usefulness, trust in the rental market, and policy perception can be important factors in guiding public housing consumption stereotypes.

**Graphical Abstract:**
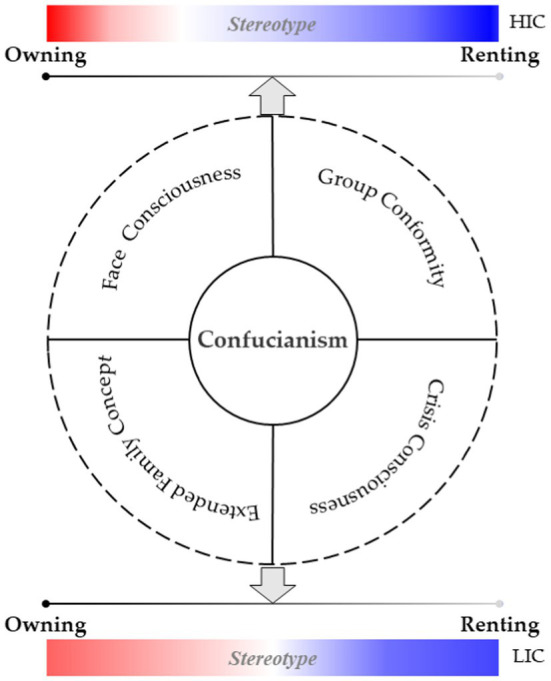


## Introduction

Since the housing reform in 1998, China’s housing sales market has been flourishing, but the rental market has been lagging behind (Ding et al., 2017). According to relevant statistics, the proportion of rental housing in developed countries such as the United States, Japan, Britain, and other countries is about 30–60% (Shi et al., 2013). However, China’s rental population is only 11.6%, much lower than that of developed countries (China Industry Information Network, 2018), which is in stark contrast to the recent decline in home ownership in the West (Clark, 2019). As a result, there has been a serious imbalance between the housing sales market and housing rental market, highlighted by the fact that “the rental market has one short leg and the sales market has one long leg.” For a long time, China’s housing rental market and sales market is not balanced, which has led to persistently high housing prices in China’s large and medium-sized cities (Guo et al., 2021), the normal consumption of young people is overcrowded by housing (Waxman et al., 2020), and the high investment in fixed assets of the real estate industry makes the development of other industries of the national economy insufficient (Wang et al., 2022).

Therefore, cultivating and developing the housing rental market is an inevitable choice to revitalize consumption and promote internal circulation under the new development pattern. Most of the existing studies on housing tenure choice are policy-oriented and focus on macro-level studies, such as the impact of economics, institutions, and socio-demographic characteristics (Chen et al., 2019). However, a large portion of the change in homeownership remains unexplained. For example, Chinese households continue to tighten their belts to buy homes despite rising house prices (Waxman et al., 2020), while the United States housing market saw homeownership rise more in 1995 and 2005 but fall more in 2015, which cannot be explained by demographics (Goodman and Mayer, 2018). The above studies are still lacking in explaining the underlying mechanisms that explain why Chinese people buy houses despite high housing prices. Neoclassical economics lacks explanatory power for the underlying behavioral logic. Therefore, it seems critical to consider other factors that influence homeownership choices. Some studies have shown that culture is an important factor influencing consumers’ housing consumption decisions (Marcén and Morales, 2020). Particularly in the Chinese cultural context, housing consumption behavior is not only a commodity-rich behavior, but it is also a socio-culturally distinct behavior. For more than 2,000 years, Confucian culture is the core of Chinese traditional culture and plays a dominant role, and its customary beliefs and values passed down from generation to generation have a subtle and profound impact on the housing consumption decisions of the Chinese public (Wu et al., 2020).

However, the existing studies have the following shortcomings. On the one hand, most of the existing studies only consider the influence of a single dimension of Confucian culture, and lack an in-depth exploration of the subdivision dimensions of Confucian housing culture. In addition, most of the existing studies have used subjective reporting methods to obtain data and have conducted empirical studies using economics and statistics to confirm the influence of Confucian culture on housing consumption behavior. However, there are some practical limitations in the existing studies, such as individuals may not be able to express their true opinions and feelings due to face, catering to policies, and other factors, such as the implicit attitude of “applause but not the audience” in their responses. In recent years, the development of cognitive neuroscience has provided objective, visual, and scientific explanations for individual consciousness and behavior (Cheng et al., 2022; Guo et al., 2022; Peng et al., 2022). Based on this, this study examines the public stereotypes of housing consumption behavior from the perspective of Confucian culture, and further analyzes the influence of the degree of Confucian cultural influence on the public’s cognitive attitude toward housing consumption, revealing the inhibiting effect of Confucian culture on housing rental demand.

Qi and Ploeger (2021) studied the influence of cultural concepts on Chinese people and found that Chinese people are very face consciousness and face consciousness. Fong (2021) found that Chinese people emphasize more on family responsibilities and obligations when comparing the differences between Chinese and Western cultures. Hofstede (1993) found that Chinese people are relatively more risk-averse and pursue long-term orientation and collectivism. Based on the above studies, this paper first summarizes the connotation of Confucian culture in Chinese society into four dimensions, including face consciousness, extended family concept, group conformity, and crisis consciousness. Then, by constructing a questionnaire using the dichotomous method, the subjects were divided into high and low impact groups from the overall and segmentation dimensions. At the end of the experiment, the participants’ explicit attitudes were measured by a self-reported questionnaire. Finally, we measured the stereotype of housing consumption by Confucian culture using the electroencephalograph (EEG) method and explored the implicit attitudes of individuals using ERPs techniques. Our study contributes to the existing literature in the following ways. First, this study extends Confucian culture-related research by considering the influence of the Confucian culture segmentation dimension. Second, this study applies physiological measures to the field of urban housing research, and uses research tools from cognitive neuroscience to validate and refine the stereotypes of rental housing among youth group, revealing the variability of cognitive perceptions and decision-making behaviors among individuals at a deeper level. It is the first time to explore the activation model of housing consumption stereotypes, which expands the related research in the field of urban housing.

The rest of this paper is organized as follows. Section “Literature Review and Research Hypotheses” reviews relevant literature and raises research hypothesis. Section “Materials and Methods” introduces experimental methods, including experimental procedures and data collection. Section “Results” presents the results of data analysis. Section “Discussion” analyzes the conclusions obtained directly from the results and gives the corresponding solutions. Lastly, Section “Conclusion and Limitations” summarizes the findings of this study and points out the limitations of existing studies.

## Literature Review and Research Hypotheses

### Influence Factors of Housing Tenure Choice

Housing purchase choice is not a simple investment or consumption decision, but a complex system event (Clark and Dieleman, 1996). Since the beginning of the research on housing purchase choice in the 1980s, the factors influencing housing tenure choice can be basically summarized as microeconomic factors, macroeconomic environment, socio-demographic characteristics, psychological factors, cultural factors, etc. From an economics perspective, microeconomic factors can be subdivided into uncertainty in residents’ income, household savings, credit status, and net household liquidable wealth (Lin and Lai, 2018), and changes in the macroeconomic environment including rising consumer prices, rising mortgage rates, subsidy regimes, and rent controls (Peng et al., 2020; Lin and Tsai, 2021). Enstrom-Ost et al. (2017) uses a two-stage instrument variable (IV) logit and probit model to study tenure choices in the Swedish housing market, which shows that financial constraints are inversely related to homeownership. Based on an online questionnaire, Liu and Li (2018) researched home purchase decisions in urban China and found that fixed income, for children’s education, solid credit, impending childbirth, and marriage are the five most important factors. From a sociological perspective, demographic characteristics can be subdivided into age, gender, marital status, education of the household head, and number of working population (Fang and Zhang, 2016). Chen (2016) based on household survey data in Guangzhou, found that housing tenure choice was positively correlated with marital status, age, and education, revealing the heterogeneity of socioeconomic status on housing tenure choice. Anderson et al. (2021) Using 2004 and 2008 panel data from the Survey of Income and Program Participation (SIPP), household secured debt, household wealth, and household income play an important role in changes in household tenure choices. Scholars such as Hochstenbach and Boterman (2017) argue that the impact of parental housing tenure on children’s intergenerational support is also not negligible. From a psychological perspective, personal subjective factors, such as property ownership preferences, can have a significant impact on housing tenure choices (Zingales, 2015). Rubaszek and Rubaszek (2021) showed the impact of psychological factors on college students’ preferences for home ownership through questionnaires at both economic and psychological levels. Foye et al. (2018) argued that homeownership provides intangible psychological benefits and is seen as a symbol of one’s social status and home property is seen as a status commodity. Rohe et al. (2013) argued that from a psychological perspective, homeownership provides homeowners with a sense of belonging and self-esteem compared to renters.

### The Influence of Culture on Housing Tenure Choice

Culture contains a set of enduring beliefs or values that permanently and profoundly influence and solidify people’s cognitive preferences and decision-making behaviors (Alesina and Giuliano, 2016). Under different cultural backgrounds, residents’ family concepts and intergenerational relationships vary widely, and their housing consumption preferences will also change accordingly, which will lead to differences in their housing tenure choice. In terms of research on culture on homeownership choices, Marcén and Morales (2020) analyzed the role of culture in determining whether an individual is a homeowner using data from first-generation immigrants arriving in the United States under the age of 6, suggesting that culture is a homeownership decision important factors. Cultural differences and policy biases affect housing rental and purchase choices (Tabner, 2016). In Germany, rental housing is of good quality and can be used as an alternative to home ownership, usually with a transition to first-time homeownership at a later time after the first birth (Mulder and Wagner, 1998). In Lagos State, socio-cultural experience of low cost residential areas in Ikorodu influenced resident satisfaction, and cultural background was found to be an important factor influencing consumer housing consumption choices (Makinde, 2015). In Asia, Wei et al. (2017) argue that marriage to buy a house is a culture, and marriage triggers demand for home buying as young men use home buying as a strategy to improve their relative position in the marriage market.

In the context of Chinese culture, Mu et al. (2021) believe that with the deepening of marketization in China, the economic and cultural importance of housing ownership has increased, and the desire of young people to own housing has also increased. Huang et al. (2020) explores the causes of high homeownership in China from a cultural perspective and constructs an empirical model to show that cultural traditions stemming from patrilineal marriage and the cultural perception of homeownership as a status symbol drive the high homeownership rate of Chinese residents. Based on the 2011 China Household Finance Survey, Wu (2020) examined the impact of Confucian culture on home ownership, and the study showed that household heads with more siblings were more likely to own a house, confirming the interdependence among family members. Currently, housing is the embodiment of culture, representing an important status and bringing various benefits (Zheng et al., 2016). Thus, rooted in traditional Chinese culture, family structure has become a trigger for entry into homeownership and has gradually evolved to make homeownership a necessary condition for marriage (Hu and Wang, 2019).

### ERP Components Relate to Stereotypes

Stereotype refers to the relatively fixed views on specific types of people or things. As a social cognitive schema, it can simplify the process of social cognitive processing and enable people to quickly form the cognition of the corresponding objects (Hilton and Hippel, 2003). In terms of their valence, stereotypes have both negative and positive aspects. Facts have proved that brand stereotype influences favorable consumer behaviors toward a brand (Japutra et al., 2021). Country of origin stereotypes affect consumers’ product evaluations (Xie et al., 2018). In short, stereotype affects all aspects of people’s lives. Interactive Memory Systems Account distinguishes explicit and implicit attitudes, revealing the processing system of different components, including cognition, emotion, and behavior (Amodio, 2019). Explicit stereotypes are based on conscious cognitive processing, and fail to account for underlying (i.e., implicit) biases (Tse and Tung, 2020). Different from explicit stereotypes, implicit stereotypes operate without conscious and controlled intentions (Devine, 1989). Implicit stereotypes are based on fast systems and emotions, making individual instinctive decisions. Thus, in implicit stereotype measures, people are required to respond spontaneously to relevant stimuli in order to examine the individual’s underlying associations with these stimuli.

Event-related potentials (ERPs) are evoked potentials elicited by stimuli, which reflect EEG signals synchronized with the presentation time of stimuli and show people’s objective cognitive and psychological behavior. With millisecond temporal resolution, ERPs technology is able to track the extremely fast processes involved in language processing, allowing real-time assessment of the neural activity of cognitive processes (Fan et al., 2021). As a result, ERPs techniques are widely used to explore the neural mechanisms behind human decision-making (Yu et al., 2022). In addition, studies have been conducted to explore the cognitive neural mechanisms of implicit stereotypes through ERPs technology. Therefore, ERPs is well-suited for exploring the existence of implicit stereotypes and their causes for excavation.

The N400 component of ERPs, first identified as a result of semantic mismatch, is an ERPs signal for semantic processing that peaks around 400 ms after stimulus onset. There is a more-prominent N400 response when the end of the sentence is semantically incongruent (relatively consistent) with the rest of the sentence (Kutas and Hillyard, 1980). In numerous studies, the N400 component has been considered as an observable indicator of stereotypes, and experimental stimuli with stronger conflict significance can induce larger N400 ERPs wave amplitudes. For example, Hou et al. (2021) used the N400 to explore public stereotypes of the recycled water end uses. Grant et al. (2020) used the N400 to explore how gender stereotypes affect the semantic processing of male (sports) and female (fashion) speaker statements. In addition, all of the above studies suggest that stereotype incongruity conditions induce greater N400 wave amplitude. Therefore, the ERPs component N400 was selected as an observational indicator in this study to investigate whether the public has implicit stereotypes about rental and home purchase consumption.

The above studies show that housing tenure choice is not just a rational behavioral decision, especially since Eastern cultures have more cultural and emotional connotations than Western cultures, which tend to be rational and materialistic. However, the existing research has the following shortcomings. First, most existing studies take homeownership preferences for granted, and the causes and processes of their formation need to be explored in depth. In addition, existing research has shown the influence of culture on housing consumption preferences, ignoring the extent of cultural influence on perceived housing consumption preferences and decision-making behavior.

### Research Hypotheses

Under the influence of Confucian culture, what are the public’s stereotypes of renting and home ownership? For a long time, Chinese people have a “rent-averse” culture and a tradition of home ownership, and a cultural perception and preference for owning property, and owning their own home is ingrained in Chinese families (Huang et al., 2015). People have always believed that owning a home is the only way to have a family, and that owning a home is the only way to feel safe and happy. At the same time, since in most cases, renting is seen by the general public as a transitional housing, it also does not allow them to reflect their status, let alone to have a good living environment and supporting living facilities and rights (Zheng et al., 2020). Therefore, people have an obsession with the idea that only when they own their own home in a city can they be considered to be rooted in that city. Based on the above analysis, the following hypotheses are proposed in this paper.

*H1a*: The public has a negative stereotype about rental housing.

*H1b*: The public has a positive stereotype of home ownership.

Face consciousness has been one of the most important ideas for Chinese people since ancient times, and it is also the core of Confucian cultural values (Wei and Jung, 2017). The love of face is a major national character of the Chinese people (Long and Aziz, 2021), which profoundly influences people’s value orientation and behavioral intentions (Xie and Shi, 2021). In addition, Chinese people have a strong desire to compare with each other. To some extent, what Chinese people buy is not a house, but a face. As a result, homeowners have higher self-confidence or enjoy higher social status (Hu and Ye, 2020). Therefore, individuals with a high face consciousness will make every possible effort to gain or maintain face. For example, in terms of consumption behavior, consumers with high face consciousness tend to buy luxury goods with scarcity characteristics, so as to reflect their social status and achieve the purpose of self-realization. In terms of face consciousness and housing consumption behavior, since renting does not reflect one’s status in the eyes of the public, groups with high face consciousness are more reluctant to rent housing. Based on the above analysis, this paper proposes the following hypotheses.

*H2a*: Face consciousness had a positive effect on negative stereotypes of renting, with greater N400 peak amplitude in the high face consciousness group (HFC Group) compared to the low face consciousness group (LFC Group).

*H2b*: Face consciousness has a positive effect on positive stereotypes of home purchase, with greater N400 peak wave in the HFC Group compared to the LFC Group.

Group conformity is an important feature of Confucian culture. Group conformity perception refers to subjective perceptions that are influenced by one’s own group or reference group and want to be consistent with that group. It is widely believed that Chinese collectivist values originate from Confucian culture, and that collectivism under Confucianism is based on interdependence, group subordination, and group behavioral norms (Liao et al., 2018). Therefore, the collectivism-oriented culture in China encourages people to follow group conformity, and individual choice intentions are easily influenced by others (Nasrin, 2008). Chinese people are very concerned about whether their behavior is consistent with the group, and there is a strong psychology that is consistent with the group behaviors (Li and Su, 2007). In the housing consumption decision, the group unanimously prompts consumers to make the decision to buy a house. Therefore, the stronger the individual’s subjective perception of group conformity, the more he or she prefers to purchase a house. Based on the above analysis, this paper proposes the following hypothesis.

*H3a*: Group conformity has a positive effect on negative stereotypes of renting, with greater N400 peak amplitude in the high group conformity group (HGC Group) compared to the low group conformity group (LGC Group).

*H3b*: Group conformity has a positive effect on positive stereotypes of home purchase, with greater N400 peak amplitude in the HGC Group compared to the LGC Group.

Chinese urban families are predominantly large, and the concept of extended family in Confucian culture is rooted in the sense of responsibility of family members who are closest to each other by blood (Liu and Li, 2018). In China, housing is not only a carrier of the family, but also provides emotional security for blood relatives under the same roof (Hu and Ye, 2020). In combination with the deep-rooted cultural desire in China, homeownership is also a necessary step in fulfilling the obligation and responsibility to provide security for the family (Fong et al., 2021). In addition, driven by China’s mother-in-law economy, home ownership is seen as a basic requirement and a major signal of marriage (Fang and Tian, 2017), and as a bond for the continuation of family inheritance. Therefore, the more individuals are influenced by family perceptions, the more they prefer to purchase a home. Based on the above analysis, the following hypotheses are proposed.

*H4a*: Extended family concept has a positive effect on negative stereotypes of renting, with greater N400 peak amplitude in the high extended family concept group (HEFC Group) compared to the low extended family concept group (LEFC Group).

*H4b*: Extended family concept has a positive effect on positive stereotypes of home ownership, with greater N400 peak amplitude in the HEFC Group compared to the LEFC Group.

Chinese have a strong sense of risk aversion and tend to make long-term plans. Homeownership offers greater financial liquidity due to secured borrowing. Meanwhile, homeowners can hedge their risk and avoid uncertainty about rent levels with nominally fixed mortgage payments (Goodman and Mayer, 2018). Homeownership not only provides a place for individuals to settle down, but also brings spiritual sense of belonging and security (Huang et al., 2015). In the long run, owning a house is a way for the Chinese to hedge against risk. Therefore, home ownership is a guarantee of financial security, and preference for buying a house is even more profound in China (Zheng et al., 2020). Based on the above analysis, the following hypotheses are proposed.

*H5a*: Crisis consciousness has a positive effect on negative stereotypes of renting, with greater N400 peak amplitude in the high crisis consciousness group (HCA Group) compared to the low crisis consciousness group (LCA Group).

*H5b*: Crisis consciousness has a positive effect on positive stereotypes of home ownership, with greater N400 peak amplitude in the HCA Group compared to the LCA Group.

Policy perception refers to individuals’ knowledge of policies and their evaluation of the effects of policy formulation, implementation, and enforcement; perceptions determine preferences, which further guide their behavior and decisions. Policy perception plays an important role in farmers’ decision making process as meaningful moderators reflecting individual attitudes, environmental influences, and social norms (Fan and Zhang, 2019). In addition, legal policy perception positively moderates the relationship between environmental intentions and behavior (Cheng et al., 2020). The higher the youth group’s knowledge and recognition of housing rental policies, the stronger the role of relevant rules in guiding their behavior, and the more likely they are to live in rental housing. Based on this, this paper proposes the following hypothesis.

*H6*: Policy perception negatively moderates the relationship between Confucian culture and housing consumption stereotype.

Perceived usefulness influences individuals’ behavioral attitudes and has an impact on behavioral intentions. Mwesiumo et al. (2020) investigated the impact of public purchasers’ attitudes toward public procurement innovation (PPI) through a self-administered questionnaire and showed that perceived usefulness is the most important factor influencing attitudes toward PPI in public procurement innovation. In addition, Chi (2018) explored external market-oriented consumer technology acceptance from the consumer’s perspective, and the study showed that perceived usefulness can influence behavioral intentions by enhancing consumers’ attitudes toward use. In the assessment of housing consumption intentions, perceived usefulness reflects the extent to which consumers perceive that the act of purchasing a home enhances various aspects of their life process, such as the ability to buy a home to settle in a household and solve their children’s education problems. Based on this, this paper proposes the following hypothesis.

*H7*: Perceived usefulness negatively moderates the relationship between Confucian culture and housing consumption stereotype.

The imbalance of housing services in China’s housing sales market and housing rental market, the fact that owned housing has more autonomy than rented housing, the lack of rental market policies and the lack of regulation. Coupled with the fact that residential income is still dominated by the growth of house prices, the increase in rent is small compared to the increase in house prices, while the different rights of renting and purchasing make the property rights have a certain impact on the equality of rights, so residents’ trust in the rental market will affect the preference for home purchase. This paper assumes that the higher the trust in the rental market, the more inclined to rent a residence. Based on this, this paper proposes the following hypothesis.

*H8*: Psychological factors negatively moderates the relationship between Confucian culture and housing consumption stereotype.

Income is an important influential factor in determining housing tenure choice (Anderson et al., 2021). Most scholars believe that households with higher lasting income as well as better stability are more likely to purchase a home and have higher requirements for housing comfort. Jorgen et al. (2005) analyze the consumer housing consumption market through an objective function of consumer utility maximization and show that consumers’ preference for ownership leads them to choose homeownership, while those with no preference for ownership are more. The results of this study show that consumers’ preference for ownership leads them to choose homeownership, while those who have no preference for ownership prefer to rent housing. Based on the above analysis, this paper proposes the following hypotheses.

*H9*: Income positively moderates the relationship between Confucian culture and housing consumption stereotype.

**Figure 1:**
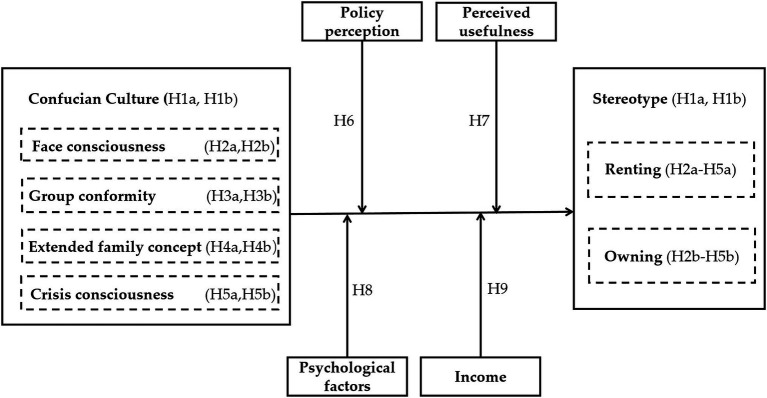
Conceptual framework.

In summary, the theoretical analysis framework of this paper is shown in Figure 1.

## Materials and Methods

### Participants and Data Collection

As the main force of housing demand, understanding why the youth group prefers home ownership among various forms of housing is conducive to alleviating the strong demand in the home purchase market in the process of implementing “houses are for living in, not for speculation.” It is also conducive to better respond to the needs of the youth group in the process of fostering rental housing and developing subsidized housing. However, there is a lack of research on the attitudes and perceptions of youth group, and the importance of homeownership to young people has been overlooked to some extent (Lindblad et al., 2017). Therefore, in this study, we selected the youth group as the study population and recruited school students as participants to explore the stereotypical housing consumption of the youth group under different degrees of Confucian cultural influence for the following reasons. First, the participants we selected are all senior graduate students or doctoral students (about 24 years old) who are about to go out of campus to look for jobs, and their decision to buy a house is also one of their most important considerations (Sun et al., 2021). In fact, according to the latest survey data from the National Bureau of Statistics of China, the average age of marriage for men and women in China is about 25^1^ years old, which is similar to the average age of the participants in this experiment. To a certain extent, there is an understanding of this research question, which can be approximated to represent the youth group. Second, this study adopts the paradigm of psychological experiments. In psychological research, considering the cheapness and the need for a high degree of cooperation in EEG experiments, many studies recruit university students as participants. In addition, in the cognitive neural experiments in the social sciences, students are also mostly selected as participants (Hou et al., 2021; Zang et al., 2021; Liu et al., 2022).

We posted an advertisement for participants on the social media WeChat public website and randomly selected participants for the experiment. Then, a total of 36 students from Xi’an University of Architecture and Technology participated in the experiment. The experimental data of six participants were excluded due to the number of trial superimposition requirements that needed to be met for each stimulus type (ensure that ERPs waveforms under each condition exceed 30 trials; Ma et al., 2021). Therefore, valid experimental data from the remaining 30 participants (11 males, 19 females; mean age: 24.17 ± 1.60 years) were recorded for analysis. All participants in the experiment were right-handed, in good health, and had normal or corrected normal vision. Indicators of the demographic characteristics of the participants are shown in Table 1. At the end of the experiment, participants were given RMB 66 as a reward. In addition, this experiment strictly adhered to the Declaration of Helsinki and its later amendments (World Medical Association, 2014). The Laboratory of Neuromanagement in Engineering of Xi’an University of Architecture and Technology approved this study. Each participant filled out a subjective questionnaire after the end of the experiment. The details of the questionnaire for this study are presented in the form of an Appendix.

**Table 1:** Demographic characteristics of the participants (*N* = 30).

Description	Items	Frequency (percentage)
Gender	Male	11 (36.7)
	Female	19 (63.3)
Age	24 or less	15 (50.0)
	24 or more	15 (50.0)
*Per capita* monthly income	3,000 or less	7 (23.3)
	3,000–5,000	15 (50.0)
	5,000	8 (26.7)
Father’s occupation	Government official	8 (26.7)
	Farmer	8 (26.7)
	Professionals	4 (13.3)
	Others	10 (33.3)
Mother’s occupation	Government official	7 (23.3)
	Farmer	9 (30.0)
	Professionals	2 (6.7)
	Others	12 (40.0)

### Stimulus Materials

The experimental material consisted of 256 stimuli, which included 16 photographs (Owning vs. Renting) × 16 words (Positive vs. Negative). The stimulus materials used in our study were determined by the following steps. First, adjectives were used to describe individuals’ attitudes toward rental and home purchase consumption. A vocabulary of 32 words (16 positive vs. 16 negative) was first constructed from the Chinese Affective Words System (CAWS), and a five-point Likert scale (1 = “extremely disagree” to 5 = “extremely agree”) was established to set the questions and distribute the questionnaire. After that, 30 respondents were recruited, who were not involved in the EEG experiment. Subsequently, they were asked to rate their individual attitudes toward rental and home purchase consumption. Then, the eight words with the highest mean scores from the positive and negative adjectives, respectively, based on the adjective scores, were selected as the stimulus words in this experiment. Finally, 16 Chinese words (eight positive and eight negative) about perception of housing consumption attitudes were selected. The Chinese words used in this study are shown in Table 2. Thus, we have four conditions of stimuli, including owning-positive, owning-negative, renting-positive, and renting-negative. Participants were provided with a keyboard and responded by pressing either the “1″ or “3″ key on the keyboard (key 1 for agree and key 3 for disagree). Before the formal experiment started, all participants were given a brief introduction about the experimental process. After they fully understood the process, the experiment started.

**Table 2:** Chinese-English translation of the lexical stimulative materials.

Positive Chinese words	English meaning	Negative Chinese words	English meaning
自在	Self-contained	约束	Restrictive
快乐	Pleasure	痛苦	Painful
安逸	Restful	压力	Stressful
满足	Satisfied	奔波	Rush about
幸福	Happiness	不幸福	Unhappy
温暖	Warm	漂泊	Vagrant
安全	Safety	危机	Crisis
安心	Comfortable	迷茫	Confusion

### Experimental Procedure

The stimulation process was presented using E-prime 3.0 programming, and the entire experiment was consisted of 256 trials. The initial eight trials were pre-experiment, and the stimulus materials in the pre-experiment were used only for practice, not in the formal experiment, to promote participants’ understanding of the experimental procedures. In order to alleviate the fatigue of the participants during the experiment, the 256 trials of the formal experiment were randomly separated into two blocks, with each block consisting of 128 trials, and the participants rested for 2 min after each experimental step of one block was completed. The size of each picture was 1,920 × 1,080 pixels.

**Figure 2:**
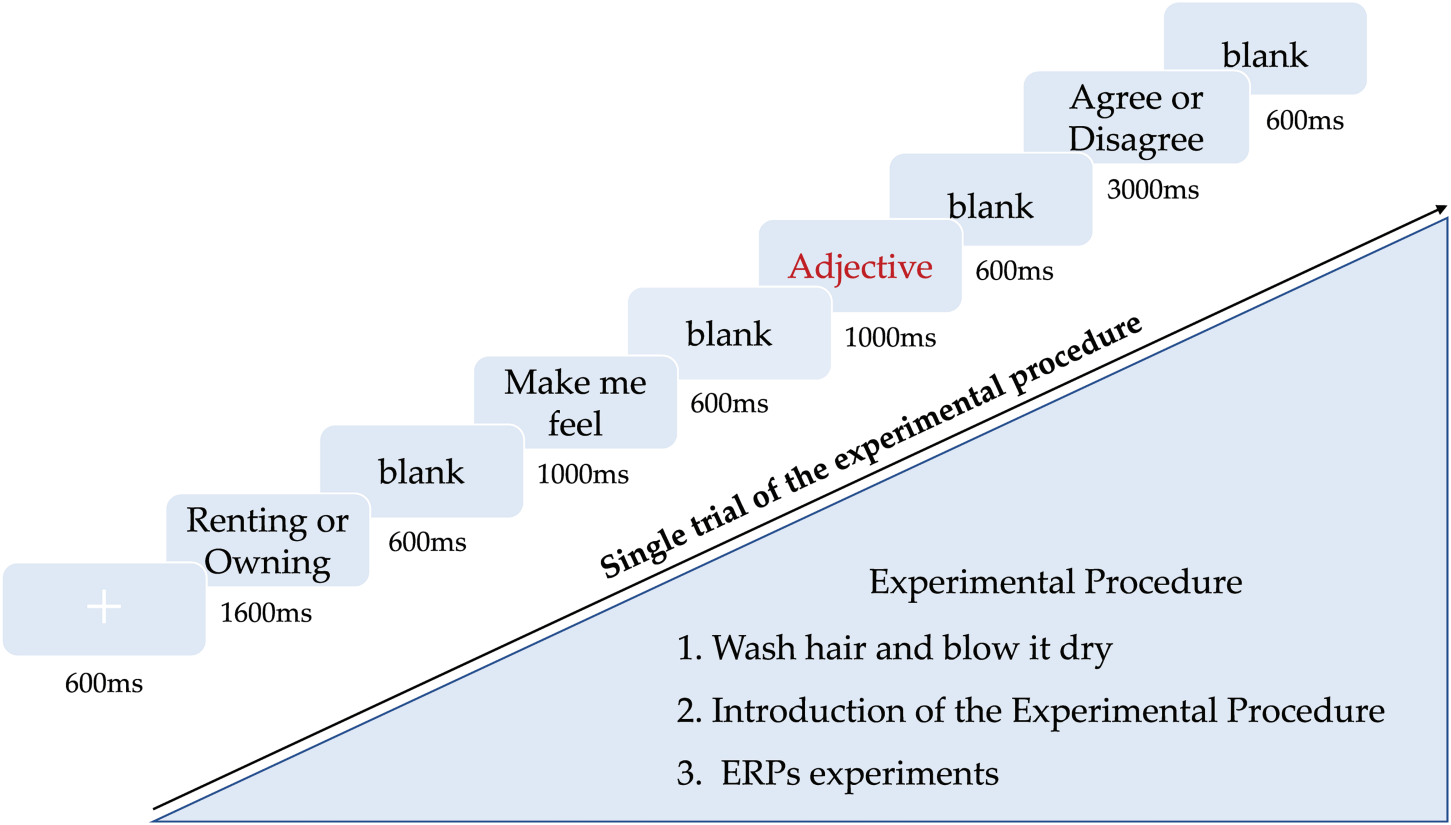
Single trial of the experimental procedure.

Prior to the experiment, the participants were told that they would need to view a series of real-life pictures with text about renting and owning a house. Next, they would observe either positive or negative adjectives, at which point participants were asked to indicate whether they agreed with the adjectives provided through a keystroke task to describe their attitudes about renting and owning a house. In the experiment, the participants sat comfortably, approximately 100 cm from a computer monitor with the stimulus material presented in the center of the screen. The experimental procedure is as follows. (i) At the beginning of each trial, a 600 ms fixation cross appeared on the screen to indicate the start of the trial. (ii) Next, a picture of a rented or purchased house was displayed for 1,600 ms. (iii) Then, a blank screen appeared for 600 ms. (iv) Then, the phrase “make me feel” appeared for 1,000 ms. (v) After that, a blank screen appeared for 600 ms. (vi) Subsequently, a positive or negative Chinese adjective was displayed for 1,000 ms. (vii) After the lexical stimulus, a blank screen was presented for 600 ms. (viii) Then, an interface asked participants to press a key (key 1 for agree and key 3 for disagree).. The notification disappeared immediately after the participant pressed the button. If the participant did not press the button within 3,000 ms, the notification page also disappeared. Each experiment takes about 1.5 h from pre-preparation to the end of the experiment. During the experiment, participants were asked to minimize eye and muscle movements. The experimental procedure and details of each trial are shown in Figure 2.

### Electroencephalography Data Recording

The electroencephalograph signals were recorded (sampling rate 1,000 Hz) using a cap consisting of 64 Ag/AgCl electrodes and a NeuroScan Synamp2 Amplifier (Neurosoft Labs, Inc.). Bilateral mastoid electrodes were selected as the reference point. The electrodes were placed 1 cm above and below the left eye to record the Vertical electrooculogram (EOG), while the horizontal EOGs was recorded from the right and left eye (1 cm from the lateral canthi). Subsequently, statistical analysis of the EOG data was performed. After a baseline correction process, artifact activity above ±100 μV per trial was excluded from the analysis. EEG recordings were then digitally filtered with a 30 Hz low-pass filter (Wang et al., 2016). Each EEG period was corrected for baseline from 200 ms before stimulation to 800 ms after stimulation onset, using the 200 ms before stimulation onset as the baseline. Finally, the mean value of event-related potentials was calculated separately for each stimulus type.

## Results

### Public Stereotypes of Housing Consumption Decision

#### Behavioral Results

Two-way repeated measures ANOVA on subjective reported data showed no significant main effect [*F*_(1,29)_ = 1.941, *p* > 0.05, *η*^2^*_p_* = 0.063] of housing tenure choice and a significant main effect of adjectives [*F*_(1,29)_ = 44.256, *p* < 0.001, *η*^2^*_p_* = 0.604]. In addition, there was an interaction between housing tenure choice and adjectives [*F*_(1,29)_ = 10.125, *p* < 0.01, *η*^2^*_p_* = 0.259]. For housing purchase, there was a statistically significant relationship between positive and negative adjectives [*F* = 49.986, *p* < 0.001, *η*^2^*_p_* = 0.633]. It was found that, after processing information in the heuristic system, the public tends to think that owning a house is self-contained, restful, warm, and comfortable.

#### Event-Related Potential Results

**Figure 3:**
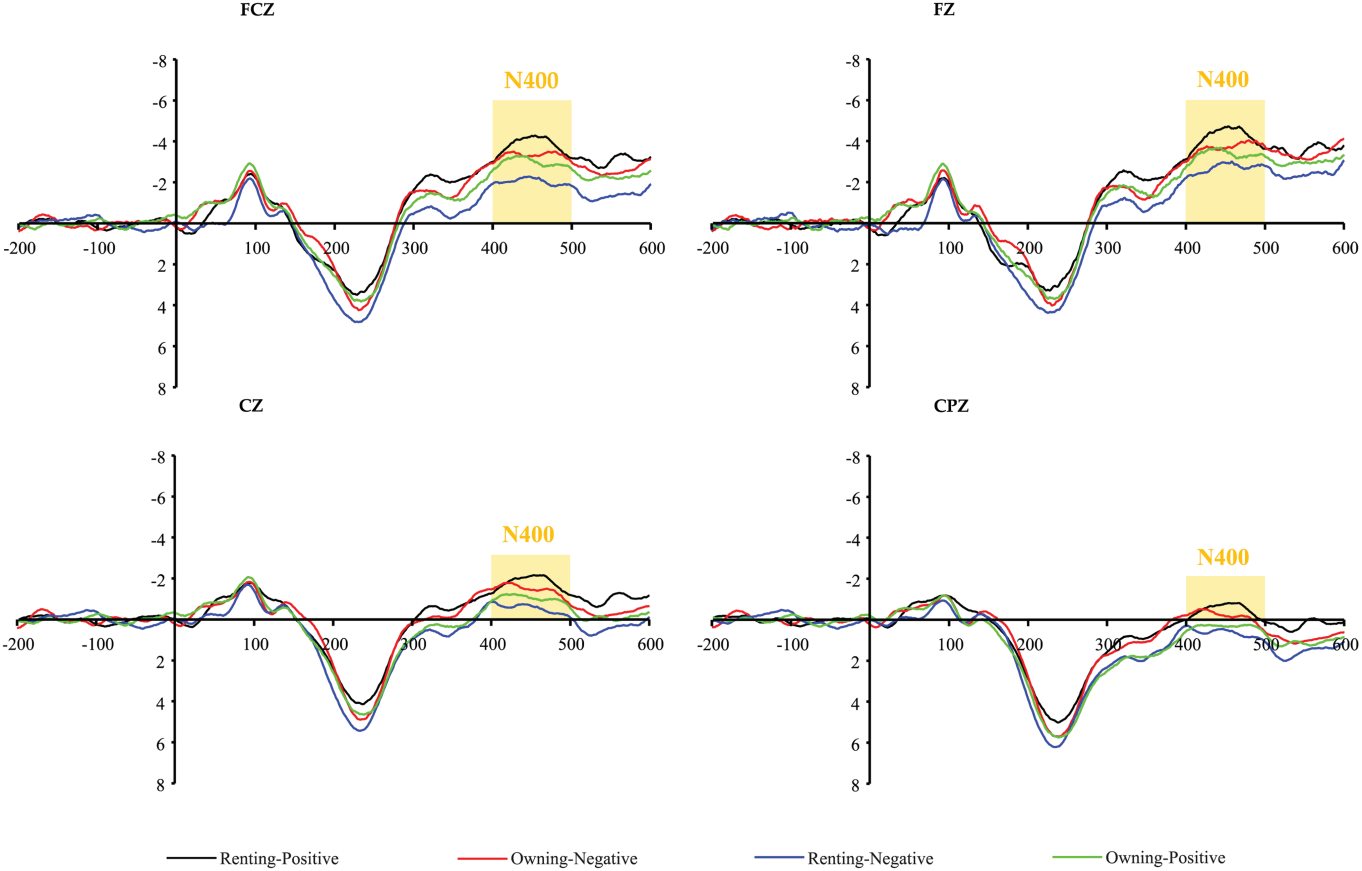
Grand-average N400 event-related potentials (ERPs) waveforms evoked by adjectives in the four electrodes (Fz, FCz, Cz, and CPz).

Semantic anomalous sentences induce N400 components at the frontal electrode sites (Seyednozadi et al., 2021). Therefore, four central electrode locations, FZ, FCZ, CZ, and CPZ, were selected to observe EEG waveforms to reflect the public’s cognitive attitudes toward renting and owning (See Figure 3). Afterward, a distinct negative wave was evoked around 400 ms of stimulus onset. Since N400 waveforms always show a peak amplitude 300–500 ms after stimulus onset, these evoked negative EEG waveforms can be considered as N400 waves (Domracheva and Kulikova, 2020). In this study, a two-way repeated measures ANOVA with peak amplitude of N400 (300–500 ms) was used to explore the public stereotypes of renting and owning a house and whether there was an interaction between the housing tenure choice (Owning vs. Renting) and adjectives (Negative vs. Positive).

**Figure 4:**
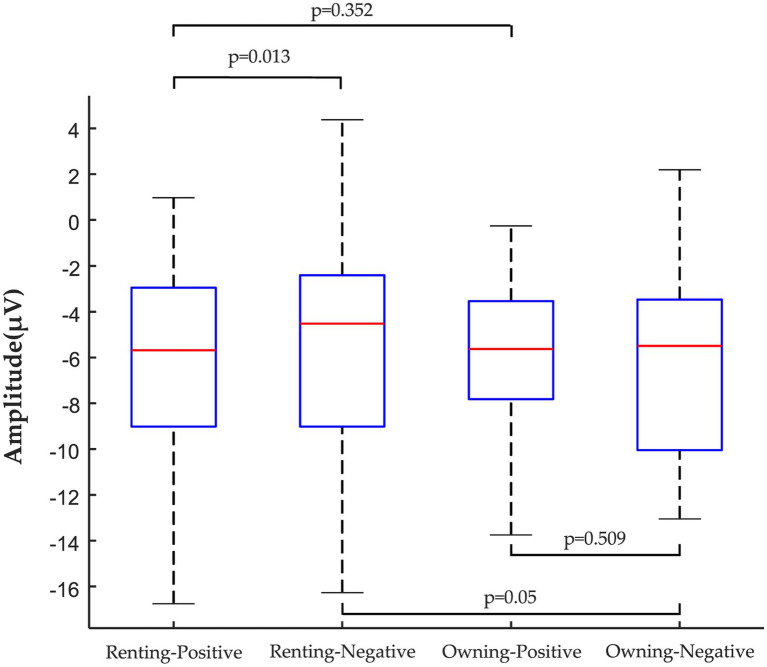
ANOVA of peak amplitude data: the comparison between the housing tenure choice and adjectives.

The observation of the four central electrode locations revealed that the target stimulus in the incongruent condition evoked a greater N400 peak amplitude in the frontal-central region compared to the target stimulus in the same condition. The ANOVA results showed no significant main effect of housing tenure choice [*F*_(1,29)_ = 0.0395, *p* > 0.05, *η*^2^*_p_* = 0.013] and adjectives [*F*_(1,29)_ = 1.156, *p* > 0.05, *η*^2^*_p_* = 0.038]. However, there was a significant interaction between housing tenure choice and adjectives [*F*_(1,29)_ = 6.323, *p* = 0.018, *η*^2^*_p_* = 0.179]. In terms of renting a house, descriptions using positive adjectives evoked greater N400 wave amplitudes in the frontal-central region than those using negative adjectives, and the peak amplitude of positive and negative target adjectives between rentals was statistically significant (See Figure 4). This shows that the public has significant negative stereotypes about rental housing consumption, Thus H1a was supported. In other words, compared to homeownership, under the cognitive processing of the fast system, the public perceives renting as constraining, stressful, unhappy, and crisis, while adjectives such as ease, comfort, warmth, and peace of mind run counter to their perceptions of renting. As a result, a larger N400 wave was generated.

### The Influence of Confucian Culture on Housing Consumption Decision

**Figure 5:**
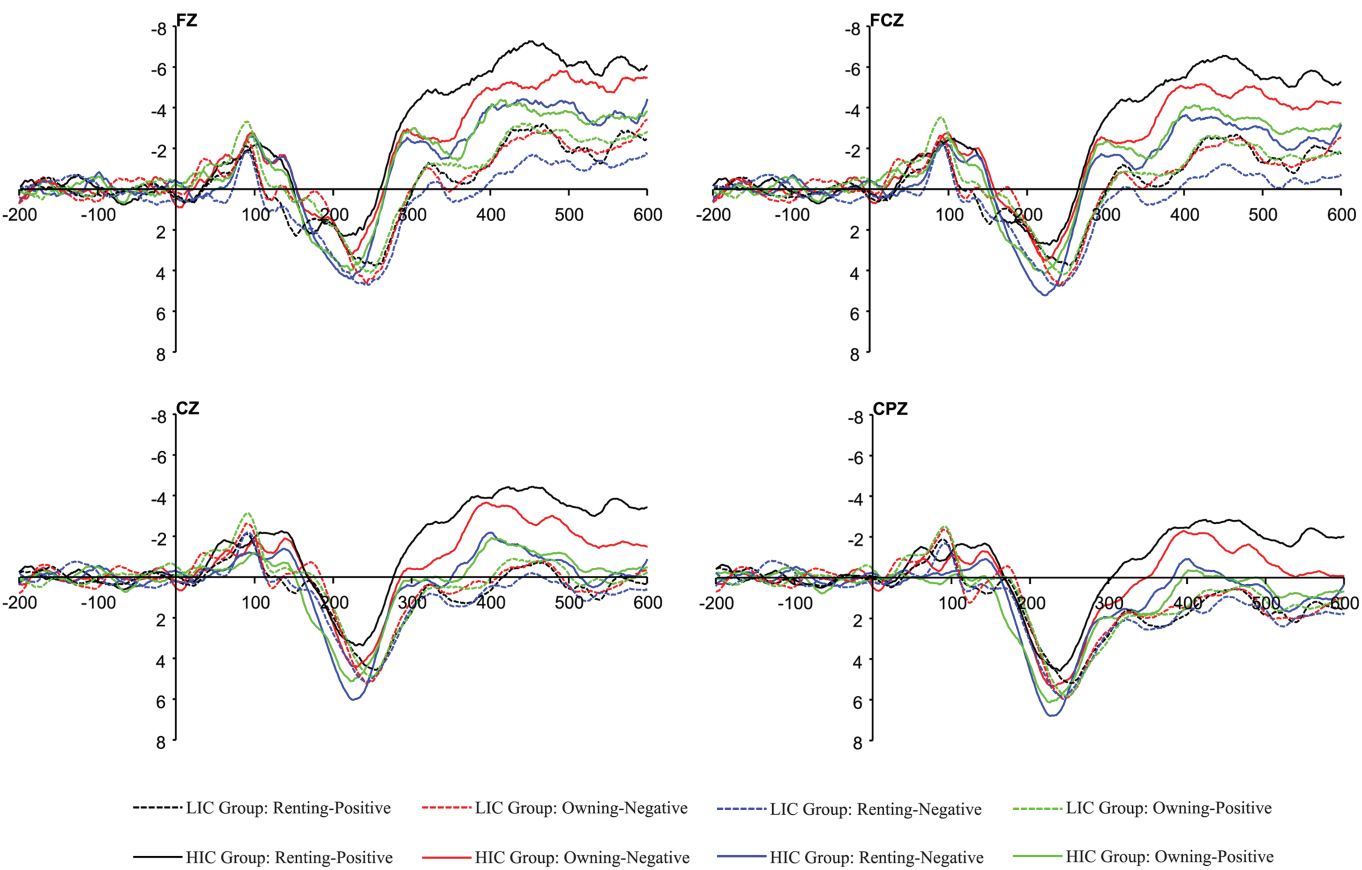
Grand-average N400 ERPs waveforms evoked by adjectives at four electrodes (Fz, FCz, Cz, and CPz) in the high Confucian culture influence (HIC) and low Confucian culture influence (LIC) Group.

First, we divided the participants into a high Confucian culture influence group (HIC Group) and a low Confucian culture influence group (LIC Group) through a questionnaire. There was statistical significance between HIC Group and LIC Group (*t* = 6.826, *p* < 0.001). The score of HIC Group was higher than that of LIC Group [(3.984 ± 0.360) vs. (3.050 ± 0.388)]. Then, a three-way repeated measures ANOVA was used to further explore the HIC Group and LIC Group stereotypes of housing consumption decisions. EEG waveforms of the four electrodes (FZ, FCZ, CZ, and CPZ) under the grouping are shown in Figure 5.

**Figure 6:**
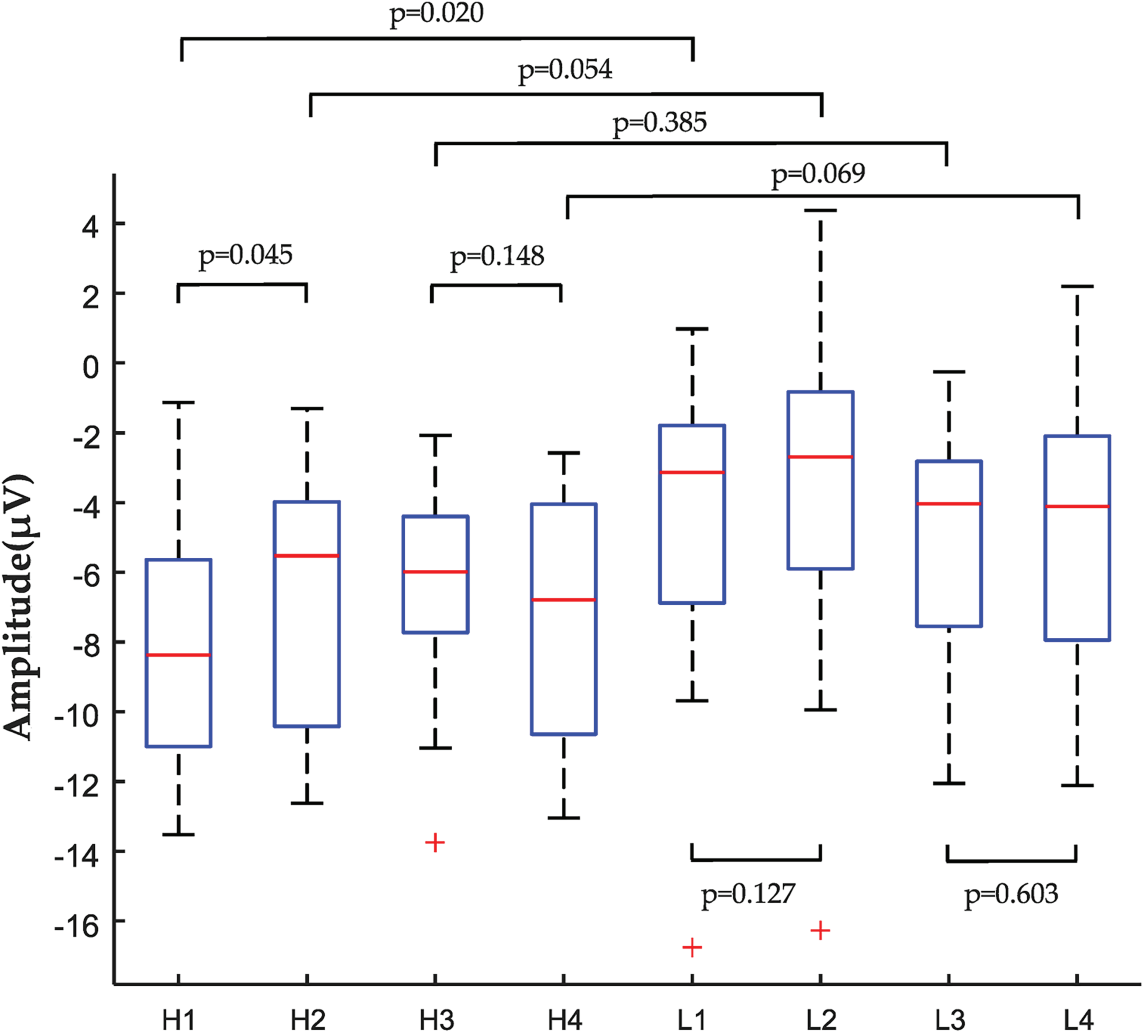
ANOVA of peak amplitude data: the comparison of the housing tenure choice with adjectives and group of high and low Confucian culture influence. (H1: Renting-Positive; H2: Owning-Negative; H3: Renting-Negative; H4: Owning-Positive; L1: Renting-Positive; L2: Owning-Negative; L3: Renting-Negative; and L4: Owning-Positive).

The ANOVA results showed that the main effects of housing tenure choice [*F*_(1,28)_ = 0.427, *p* > 0.05, *η*^2^*_p_* = 0.015] and adjectives [*F*_(1,28)_ = 1.138, *p* > 0.05, *η*^2^*_p_* = 0.039] were not significant, and the main effect of the degree of Confucian culture was significant [*F*_(1,28)_ = 4.447, *p* = 0.044, *η*^2^*_p_* = 0.137]. In terms of different Confucian cultural influence groups, a more negative stereotype of renting exists in the HIC Group compared to the LIC Group [*F*_(1,28)_ = 6.109, *p* = 0.02, *η*^2^*_p_* = 0.179], as shown in Figure 6. In other words, the more influenced the Chinese public is by Confucian culture, the more pronounced the negative stereotypes of renting consumption. Ultimately, the differences in decision-making about housing consumption choices among different Confucian culture-influenced groups were examined. The housing consumption decision data revealed that there are differences in the housing consumption decision-making behavior of the Chinese public due to different levels of Confucian cultural influence [*Z* = −2.408, *p* = 0.016]. This study shows that the housing consumption choices of the LIC group are more rational and balanced compared to the high Confucian culture influence group.

### The Influence of Confucian Culture Subdivision Dimension on Housing Consumption Stereotype

To further investigate the influence of each of the four dimensions of Confucian housing culture on the public’s housing consumption stereotypes, a three-factor repeated measures ANOVA was used. The four Confucian housing culture influence degree groupings were statistically significant (see Table 3), with all high culture influence groups scoring higher than the low culture influence groups.

**Table 3:** Confucian culture scores under the four sub-dimensions.

Confucian culture		Mean value	*t*	*p*
Face consciousness	HFC group	4.067 ± 0.537	7.101	<0.001
	LFC group	2.600 ± 0.594		
Group conformity	HGC group	3.868 ± 0.451	7.190	<0.001
	LGC group	2.311 ± 0.707		
Extended family concept	HEFC group	4.533 ± 0.353	5.879	<0.001
	LEFC group	3.490 ± 0.589		
Crisis consciousness	HCA group	4.289 ± 0.451	7.199	<0.001
	LCA group	2.979 ± 0.541		

The ANOVA results showed that the main effect of face consciousness was not significant in the renting-positive condition, and the difference in N400 peak amplitude data between the high and low face consciousness groups was not statistically significant (*p* > 0.05). In other words, the negative stereotypes of rental consumption among the youth group are not influenced by the face consciousness. The main effect of group conformity was significant (*F* = 5.528, *p* = 0.026), and there was a significant difference in N400 peak amplitude data between high and low groups of group conformity (*F* = 7.602, *p* = 0.01), Supporting H3a. The degree of influence of group conformity positively affects negative stereotypes of rental housing among youth group. The main effect of extended family concept was significant (*F* = 14.160, *p* = 0.001), with a significant difference in N400 peak amplitude data between groups with high and low extended family concept (*F* = 20.463, *p* < 0.001), Supporting H4a. This suggests that the degree of influence of extended family concept positively affects negative stereotypes of rental housing among youth group. The main effect of crisis consciousness was not significant, and the difference in N400 peak amplitude data between high and low crisis consciousness groups was not statistically significant (*p* > 0.05). There was no statistically significant difference in N400 peak amplitude data between the high and low face conscious groups in the owning-negative condition (*p* > 0.05). No significant difference in N400 peak amplitude data between groups with conformity high and low levels. There is a significant difference in the peak amplitude of N400 between the groups with high and low extended family concept (*F* = 4.886, *p* = 0.035), Supporting H4b. In addition, There is a significant difference in the N400 peak amplitude data between the groups with high and low crisis consciousness (*F* = 5.969, *p* = 0.021), Supporting H5b. The study found that group conformity, extended family concept, and crisis consciousness had significant effects on housing consumption stereotypes. Table 4 shows the average peak values and SE of N400 under the influence groups of face consciousness, group conformity, extended family concept, and crisis consciousness.

**Table 4:** The average peak value and SE of N400 for Confucian culture under the four sub-dimensions.

Confucian culture		N400			
		Renting-Positive		Owning-Negative	
		Average peak value	*p*	Average peak value	*p*
Face consciousness	HFC group	−6.729 ± 3.744	0.640	−5.731 ± 3.844	0.547
	LFC group	−5.962 ± 5.036		−6.637 ± 4.273	
Group conformity	HGC group	−9.335 ± 4.387	0.010	−7.494 ± 3.722	0.074
	LGC group	−4.357 ± 3.461		−4.874 ± 3.992	
Extended family concept	HEFC group	−9.142 ± 3.778	<0.001	−7.708 ± 3.683	0.035
	LEFC group	−3.549 ± 2.944		−4.661 ± 3.864	
Crisis consciousness	HCA group	−7.765 ± 3.036	0.078	−7.841 ± 3.439	0.021
	LCA group	−4.926 ± 5.115		−4.528 ± 3.970	

Based on multiple regression for moderating effect analysis, the interaction effect between policy perception and crisis consciousness was significant (*R*^2^ = 0.3947, ∆*R*^2^ = 0.1248, *p* = 0.0287). In other words, higher policy perception and agreement mitigated the effect of crisis consciousness on negative attitudes toward rental housing. The interaction effect between perceived usefulness and extended family perception was significant (*R*^2^ = 0.5426, ∆*R*^2^ = 0.0758, *p* = 0.0479). That is, low levels of perceived usefulness alleviated the negative impact of extended family concept on rental housing stereotype. The interaction effect between perceived usefulness and crisis consciousness was significant (*R*^2^ = 0.5033, △*R*^2^ = 0.0875, *p* = 0.0419). In other words, low levels of perceived usefulness mitigated the negative impact of crisis consciousness on rental housing stereotype. The interaction effect between psychological factors and group conformity was significant (*R*^2^ = 0.3710, △*R*^2^ = 0.1078, *p* = 0.0445). In other words, higher trust in the rental market mitigated the effect of group conformity awareness on negative attitudes toward rental housing. The interaction effect between psychological factors and group conformity was significant (*R*^2^ = 0.2346, △*R*^2^ = 0.1436, *p* = 0.0362). That is, higher rental market trust has weakened the group conformity awareness of over-preference for homeownership. The interaction effect between psychological factors and extended family concept was significant (*R*^2^ = 0.4454, △*R*^2^ = 0.1682, *p* = 0.0093). That is, higher trust in the rental market alleviated the effect of extended family concept on negative attitudes toward rental housing. The moderating effects between the remaining two variables were not significant.

## Discussion

In this study, the ERPs method was used to carry out an EEG experiment, and the N400 ERPs components representing the implicit attitude of the housing consumption stereotype were collected and counted. Specifically, as follows, the public’s cognitive attitudes and decision-making behaviors toward renting and owning a house are first explored at both implicit and explicit levels, and the stereotypes of housing consumption among the youth group are verified at the general level. After that, the differences in housing consumption stereotypes between HIC Group and LIC Group were compared. Then, the differences in the influence of housing stereotypes were explored from the high and low groups of the four dimensions of Confucian housing culture in comparison. Overall, the degree of influence of Confucian culture has a positive impact on the public’s housing consumption stereotypes. In terms of the sub-dimensions of Confucian culture, group conformity, extended family concept, and crisis consciousness are important influences on housing consumption stereotypes. Finally, significant factors affecting housing consumption were selected for moderating effect analysis, and the study showed that policy perception, psychological factor, and perceived usefulness can significantly moderate housing consumption stereotypes.

In terms of renting, ERPs data show that description with positive adjective can induce more obvious N400 amplitude. In other words, the public believes that renting is restrictive, stressful, unhappy, and crisis. Currently, we can start by breaking through these ideas to weaken the negative public attitudes toward renting. First of all, the state advocates that the social status of renting and owning a house is equivalent, owning a house is not a symbol of social status and financial ability, there is no need to purchase a house for face, renting is also a way to enjoy life. In addition, the government strengthens the supply of small-scale, low-rent rental housing suitable for young people and new citizens, so as to achieve good and stable rent, and effectively enhance residents’ sense of well-being and security.

Compared with LIC group, HIC group exists a more negative stereotype of renting. In addition, as previous studies have shown, there are differences in the decision-making behavior of the public due to their different levels of influence by Confucian culture (Xiong and Wei, 2020; Ge et al., 2021). The housing consumption decision-making data show that compared with HIC Group, LIC Group is more balanced and rational in renting and purchasing housing consumption decisions. This may have the following reasons. On the one hand, under the influence of the individual trend, some urban youth values and behavior gradually began to change, rent more easily accepted. On the other hand, the LIC group will choose housing consumption based on its own specific circumstances (e.g., income, family scale, age, etc.). Guiding the HIC group to gradually shift to the LIC group and weakening the irrational demand for home purchase will help the development of China’s housing rental market and build a housing gradient consumption model.

There was no significant difference in the N400 peak amplitude data between the high and low impact groups of face consciousness in both the owning-negative and renting-positive conditions. This may be due to the fact that the participants in this experiment represent the contemporary Chinese youth group, with generally higher education levels, which improve their ability to accept new ideas (including new policies and consumption forms), and their living concepts are more independent and cutting-edge (Zheng et al., 2019). Conversely, extended family concept played a significant role in both owning-negative and renting-positive conditions. As recognized in earlier literature, our housing tenure preferences are culturally mediated and socially constructed (Murie and Forrest, 1980), and identified that within-family socialization played a crucial role in this (Henretta, 1984). Interestingly, this study found that crisis consciousness only had a significant effect on the positive owning stereotype, whereas group conformity only had a significant effect on the negative renting stereotype. This may be because Chinese people generally have a sense of urgency. Housing ownership provides housing stability and reduces housing risks by protecting families from future rent increases, which is also a way for Chinese people to avoid risks (Bostic and Lee, 2008). In the cognitive concept of Chinese public, group conformity drives the strong psychology of individual existence and group behavior, strengthens the individual’s obsession with housing purchase, and then produces a more negative cognitive attitude toward rental housing.

The results of the moderating effect analysis indicate that policy perception, perceived usefulness, and psychological factors play a significant moderating role on housing consumption stereotypes. By vigorously publicizing the policy subsidies for the rental market to increase public awareness of macro policies, enhancing life confidence to reduce the impact of crisis consciousness, and promoting a new living culture to improve residents’ attitudes toward rental housing, the government can achieve the goal of restraining excessive expansion of housing demand. When we realize that public rejection of rental housing can be transformed by undermining the public’s perceived usefulness of the housing services that come with homeownership. In this regard, the state should gradually eliminate the differences between renters and owners in public service rights (e.g., granting tenants’ children the right to attend school nearby, permission to settle in rented houses, and access to public services such as health care and social security, etc.), and enhance the substitutability between the housing rental market and sales market (Zhang and Chen, 2018). By enhancing public trust in the rental housing market, the public’s perceived rejection of rental housing is gradually weakened. Therefore, the government should accelerate the formulation and implementation of regulations related to the rental and sale policies, increase financial subsidies and tax relief for rental housing, and strengthen the supervision and management of the rental market (Guo et al., 2021). However, there is no significant moderating effect between income and the dimensions of Confucian culture, probably because traditional cultural perceptions have made the Chinese public’s perceptions of housing consumption deep-rooted and its influence more far-reaching. Therefore, when the government vigorously develops the housing rental market, it also needs to transform the public’s perceptions of housing consumption by means of other policies that govern such cultural perceptions; no amount of policy can achieve the desired effect if it stays in the consciousness and philosophy of the past.

## Conclusion and Limitations

### Conclusion

Since ancient times, under the influence of Confucian culture, the proportion of urban households in China who rent their homes is generally low, and the housing consumption model of “preferring to own a house rather than rent one” is still common, and the demand for housing rental has not yet been effectively released. The public’s attitude toward renting and purchasing directly affects their housing consumption decision-making behavior. Therefore, it is important to study public attitudes toward renting and owning, and to guide and change them in order to finally realize the Rent and Purchase system. This study explores public stereotypes of housing consumption decisions by designing a human-factor experiment on public stereotypes of renting and homeownership consumption, and draws the following conclusions.

First, in general, describing rental housing with positive adjectives elicited significant negative brain waves between 400 and 500 ms compared to negative adjectives. That is, the public holds negative implicit stereotypes of rental consumption. However, the subjective report data suggest that the public holds positive outward stereotypes about home purchase consumption. The data on housing consumption decisions reveal that there are differences in the housing consumption decision-making behaviors of the Chinese public, influenced to varying degrees by Confucian culture.

Second, for groups with different degrees of Confucian cultural influence, the HIC Group exhibits a greater amplitude of N400 for rental housing consumption, and the negative stereotype of rental housing consumption deepens as the public’s degree of Confucian cultural influence increases.

Third, by subdividing the four dimensions of Confucian culture, the study showed that as the degree of cultural influence increased, group conformity, the extended family concept, and crisis consciousness significantly influenced housing consumption stereotypes, while there was no significant difference in the influence of face consciousness on housing consumption stereotypes.

Fourth, this study also considers other factors that may influence Confucian culture on housing consumption stereotypes. The moderating effect analysis indicates that perceived usefulness, psychological factors, and policy perception can be important factors in guiding the public’s stereotype of housing consumption, while the moderating effect of income on the stereotype of housing consumption is not significant.

### Limitation and Future Research

Although this study provides valuable and detailed insights into public stereotypes about housing consumption decisions, there are limitations to the results.

In particular, it must be noted that this paper explores public stereotypes of housing consumption from a Confucian cultural perspective, and both the aggregate and subgroup levels suggest that Confucian culture influences the public’s housing consumption decision-making behavior. However, there are deeper reasons that need to be explored. For example, to what extent does Confucian culture contribute to the Chinese public’s “preferring to own a house rather than rent one” housing consumption model? In future studies, we will explore the extent to which multiple factors influence the stereotype of “preferring to own a house rather than rent one” from multiple perspectives.

In addition, on the one hand, the Confucian housing culture scale defined and developed in this study is not fully representative of the Confucian housing culture as a whole. On the other hand, due to the limitations of the research equipment and conditions, the participants in this study were all students of Xi’an University of Architecture and Technology, and the research sample was somewhat limited.

## Data Availability Statement

The raw data supporting the conclusions of this article will be made available by the authors, without undue reservation.

## Ethics Statement

The studies involving human participants were reviewed and approved by Laboratory of Neuromanagement in Engineering of Xi’an University of Architecture and Technology. The patients/participants provided their written informed consent to participate in this study.

## Author Contributions

MY, XG, and HF contributed to conception and design of the study. XL, MY, and XG wrote the first draft of the manuscript. BC performed the statistical analysis. All authors contributed to the article and approved the submitted version.

## Funding

This study was funded by the National Natural Science Foundation of China (Grant No. 72001167), Social Science Foundation of Shaanxi Province (China, Grant No. 2020ZDWT18) and the Key Research and Development Plan of Shaanxi Province (China, Grant No. 2018ZDCXL-SF-03-04).

## Conflict of Interest

The authors declare that the research was conducted in the absence of any commercial or financial relationships that could be construed as a potential conflict of interest.

## Publisher’s Note

All claims expressed in this article are solely those of the authors and do not necessarily represent those of their affiliated organizations, or those of the publisher, the editors and the reviewers. Any product that may be evaluated in this article, or claim that may be made by its manufacturer, is not guaranteed or endorsed by the publisher.

## Footnotes

^1^ http://www.stats.gov.cn/tjsj/pcsj/rkpc/6rp/indexch.htm

## Appendix

The specific contents of the questionnaire.

**Table:**

Level	Dimension	Indicator
Demographic characteristics	Gender	Male
		Female
	Age	24 or less
		24 or more
	*Per capita* monthly income	3,000 or less
		3,000–5,000
		5,000
	Father’s occupation	Government official
		Farmer
		Professionals
		Others
	Mother’s occupation	Government official
		Farmer
		Professionals
		Others
Confucian culture	Face consciousness	I think buying a house is a status symbol
		Not owning a house is not an honorable thing
		Buying a house makes me feel good
	Group conformity	Most of the surrounding relatives/friends/colleagues own a house
		Most of my relatives/friends/colleagues around me think I should own a house
		Most of the relatives/friends/colleagues around think that owning a house is a basic conditions of life
	Extended family concept	Owning a home better nurtures and educates future generations
		Owning a home can make life better for your family
		Owning a house can give future generations a material basis for growth
	Crisis consciousness	Owning a house can ease my work-life worries
		Owning a house makes my life easier
		I think owning a home makes me feel happier
Moderator variable	Perceived usefulness	Owning a home and being able to choose the style of decoration I like drives my preference for buying a home
		Owning a home avoids having to move a lot and makes me prefer to buy a home
		I think rental housing will limit the future compulsory education of children
	Psychological factor	I think it is difficult to protect the rights of renters and landlords in the current rental market
		I have little trust in the Xi’an rental market
		I think the rental market in Xi’an is not well-served
	Policy perception	The understanding of “rent and purchase”housing system
		The attitude of “rent and purchase” housing system which guide housing gradient consumption
		The understanding of Xi’an City Talent Settlement Rental Subsidy Policy
Subjective attitudes toward housing consumption		I have a positive attitude toward renting a house (e.g., I think renting is more cost effective, a way to enjoy life, etc.)
		I have a negative attitude toward renting a house (for example, I think rented housing is constraining, unhappy, etc.)
		I have a positive attitude toward owning a house (e.g., I think home ownership is warm, comfortable, and satisfying, etc.)
		I have a negative attitude toward owning a house (e.g., I think it is not cost effective to buy a house, there is no need to cut back on other spending to buy a home, etc.)
Housing consumption decision		Owning a house
		Renting a house
